# Molecular Characterization of the RNA-Binding Protein Quaking-a in *Megalobrama amblycephala*: Response to High-Carbohydrate Feeding and Glucose/Insulin/Glucagon Treatment

**DOI:** 10.3389/fphys.2018.00434

**Published:** 2018-04-24

**Authors:** Hua-Juan Shi, Wen-Bin Liu, Chao Xu, Ding-Dong Zhang, Bing-Ke Wang, Li Zhang, Xiang-Fei Li

**Affiliations:** Key Laboratory of Aquatic Nutrition and Feed Science of Jiangsu Province, College of Animal Science and Technology, Nanjing Agricultural University, Nanjing, China

**Keywords:** RNA-binding protein, quaking-a, gene cloning, transcriptional analysis, glucose metabolism, *Megalobrama amblycephala*

## Abstract

The RNA-binding protein quaking-a (Qkia) was cloned from the liver of blunt snout bream *Megalobrama amblycephala* through the rapid amplification of cDNA ends method, with its potential role in glucose metabolism investigated. The full-length cDNA of *qkia* covered 1,718 bp, with an open reading frame of 1,572 bp, which encodes 383 AA. Sequence alignment and phylogenetic analysis revealed a high degree of conservation (97–99%) among most fish and other higher vertebrates. The mRNA of *qkia* was detected in all examined organs/tissues. Then, the plasma glucose levels and tissue *qkia* expressions were determined in fish intraperitoneally injected with glucose [1.67 g per kg body weight (BW)], insulin (0.052 mg/kg BW), and glucagon (0.075 mg/kg BW) respectively, as well as in fish fed two dietary carbohydrate levels (31 and 41%) for 12 weeks. Glucose administration induced a remarkable increase of plasma glucose with the highest value being recorded at 1 h. Thereafter, it reduced to the basal value. After glucose administration, *qkia* expressions significantly decreased with the lowest value being recorded at 1 h in liver and muscle and 8 h in brain, respectively. Then they gradually returned to the basal value. The insulin injection induced a significant decrease of plasma glucose with the lowest value being recorded at 1 h, whereas the opposite was true after glucagon load (the highest value was gained at 4 h). Subsequently, glucose levels gradually returned to the basal value. After insulin administration, the *qkia* expressions significantly decreased with the lowest value being attained at 2 h in brain and muscle and 1 h in liver, respectively. However, glucagon significantly stimulated the expressions of *qkia* in tissues with the highest value being gained at 6 h. Moreover, high dietary carbohydrate levels remarkably increased plasma glucose levels, but down-regulated the transcriptions of *qkia* in tissues. These results indicated that the gene of blunt snout bream shared a high similarity with that of the other vertebrates. Glucose and insulin administration, as well as high-carbohydrate feeding, remarkably down-regulated its transcriptions in brain, muscle and liver, whereas the opposite was true after the glucagon load.

## Introduction

Carbohydrates are regarded as the cheapest energy source for aquatic animals due to their rich availability and relatively low cost. The incorporation of this nutrient in diets could improve the physical quality of feed, promote the growth performance of aquatic species and spare proteins from being used as energy (Hemre et al., 2002). Of greater physiological importance is its potential to provide metabolic intermediates for the synthesis of various biologically compounds (Wilson, 1994). However, it is generally acknowledged that fish have a poor capability to utilize glucose for energy purposes compared with mammals (Polakof et al., 2012). Generally, most fish species (especially carnivorous ones) have an impaired glucose tolerance and often display a prolonged postprandial hyperglycemia after a glucose load or the intake of high-carbohydrate diets (Moon, 2001). To date, the underlying mechanisms for the sluggish glucose intolerance and poor carbohydrate utilization in fish are still barely understood, although several hypotheses have been proposed, such as the higher potential of amino acids compared with glucose as insulinotropins, few insulin receptors, a poor inhibition of postprandial gluconeogenesis, a low capacity of *de novo* lipogenesis to store excess glucose, etc. (Enes et al., 2009). Recently, researchers have extended this notion to post-transcriptional gene regulation in fish in order to get some enlightenment (Craig et al., 2014). Accordingly, certain progress has been made in understanding the potential roles of post-transcriptional regulators in metabolically active and insulin-sensitive tissues (Miao et al., 2017). This suggested that the post-transcriptional regulation might be closely implicated in the intermediary metabolism and cellular homeostasis in fish. Considering this, investigations regarding the molecular characterization and nutritional regulation of these post-transcriptional regulators will undoubtedly facilitate our understanding of the carbohydrate utilization by fish.

To date, various molecules have been demonstrated to be involved in the post-transcriptional regulation of gene expression, mainly including microRNAs (miRNAs) and RNA-binding proteins (RBPs) (Lobbardi et al., 2011). Within this latter category, quaking (QKI) proteins are evolutionarily conserved RBPs, belonging to the STAR (signal transduction and activation of RNA metabolism) family (Vernet and Artzt, 1997). QKI contains a single K-Homology (KH) motif, a prevalent RNA-binding domain that generally recognizes and binds a specific sequence in the 3′-UTR (untranslated region) of the target mRNAs (Lobbardi et al., 2011). Through this, QKI participates in the post-transcriptional mRNA processing, including spliceosomal complex formation, pre-RNA splicing, and mRNA export, stability, localization, and translation (Galarneau and Richard, 2005). Until now, three isoforms of *QKI* (namely *QKI5*, *QKI6*, and *QKI7*) have been characterized in mammals (Ebersole et al., 1996). These isoforms are distinguished by both the size of the molecules and the C-terminal amino acid sequence. In the past few decades, QKI has been identified to regulate diverse biological processes, such as organogenesis, cell differentiation, embryonic development and major depressive disorder (Klempan et al., 2009; Artzt and Wu, 2010; Radomska et al., 2016). However, its implications in the intermediary metabolism still remain unclear. Recently, QKI has been linked to the pathogenesis and pathophysiology of some metabolic diseases (especially the type 2 diabetes and obesity) in mammals, highlighting its important roles in normal physiology and metabolic disorders. For example, the knockdown of QKI resulted in compromised gluconeogenesis and fatty acid oxidation, which is related to the steatosis and glucose intolerance in obese mice (Lu, 2014). In addition, QKI has been demonstrated to control the adaptive hepatic energy metabolism under fasting conditions through deacetylation (Lu, 2014). However, the aforementioned information are generally derived from mammals, literature concerning the molecular and functional analysis of *qki* in aquatic animals is still quite limited. To date, to our best knowledge, the piscine paralogs of *qki* have been characterized only in zebrafish (*Danio rerio*), which comprises three homologs of *qki* (namely *qkia, qki2* and *qkib*) corresponding to those of mammals (Radomska et al., 2016). In addition, their potential roles in both embryo and muscle development have also been confirmed, suggesting that *qki* might be a key regulator of biological processes in fish (Tanaka et al., 1997; Lobbardi et al., 2011; Radomska et al., 2016). However, their implications in metabolic processes are still barely understood, as warrants further studies.

Blunt snout bream *Megalobrama amblycephala* is one of the most important economic freshwater fish in China with a worldwide distribution. In recent years, the need to maximize the profit inevitably leads to a high incorporation of carbohydrates in its feed. However, high dietary carbohydrate levels might induce metabolic disorders of this species, as consequently results in the increasing outbreaks of fatty liver disease (Prathomya et al., 2017; Prisingkorn et al., 2017). This emphasizes the urgent need to investigate the intermediary carbohydrate metabolism of this species, which unfortunately is still barely understood. Recently, a study has identified the miRNAs involved in the glucose metabolism of this species, highlighting the pivotal roles of post-transcriptional regulation in the intermediary carbohydrate metabolism of this species (Miao et al., 2017). However, such information for RBPs (a key post-transcriptional regulator) is still unavailable. Therefore, the present study was conducted to (1) clone the full-length cDNA of the RNA-binding protein *qkia* from the liver of *M. amblycephala*; (2) to determine its tissue distribution; (3) to illuminate the tissue responsiveness to glucose, insulin and glucagon load, respectively, in the metabolically active and insulin-sensitive tissues; (4) to investigate its transcriptional response to the long-term feeding of high-carbohydrate diets.

## Materials and Methods

### Ethics Statement

The use of experimental animals in the present study has been approved by the Animal Care and Use Committee of Nanjing Agricultural University (Nanjing, China). All experimental procedures involving animals were conducted following the Guidelines for the Care and Use of Laboratory Animals in China.

### Fish Culture

Blunt snout bream (average weight: 42.6 ± 0.4 g) were obtained from the National Fish Hatchery Station in Yangzhou (Jiangsu, China). Before the experiment, fish were kept in 10 indoor tanks (200 L each) at a stocking density of 25 fish per tank for 1 month to acclimate to the experimental conditions by feeding a commercial diet, which contains 32% protein and 31% nitrogen-free extract (Shuaifeng Feed Co., Ltd., Nanjing, Jiangsu province, China). Fish meal, soybean meal, rapeseed meal and cottonseed meal served as protein sources in this feed, whereas wheat bran and middlings were both adopted as the main carbohydrate sources. During this period, water temperature ranged from 26 to 28°C, pH varied from 7.1 to 7.4, and total ammonia-nitrogen was maintained below 0.2 mg/L. Then, fish were starved for 24 h, and were later divided into three parts: one for cDNA cloning and the determination of *qkia* transcriptions in various tissues, one for the glucose tolerance test (GTT) and the other for the insulin tolerance test (ITT).

### Total RNA Extraction

Four fish were randomly selected and rapidly anesthetized in diluted MS-222 (tricaine methanesulfonate, Sigma, United States) at the concentration of 100 mg/L. Then, they were sampled for 10 organs/tissues, including white muscle, liver, intestine, mesenteric adipose tissue, heart, kidney, brain, spleen, gill, and eye. The samples were kept at -80°C until analysis.

Total RNA was extracted from the samples aforementioned using Trizol reagent (Invitrogen, Carlsbad, CA, United States). RNA samples were treated by RQ1 RNase-free DNase (Takara Co. Ltd., Japan) to eliminate genomic DNA contamination. Its quantity and purity was determined by spectrophotometric analysis (the 260/280 nm ratio) and electrophoresis (1.0% formaldehyde denaturing agarose gels).

### cDNA Synthesis and 3′ and 5′ RACE

Reverse transcription was conducted using the total RNA from the liver of blunt snout bream as template and oligo(dT)18 as primer using an AMV First Strand cDNA Synthesis Kit (GeneCopoeia, Rockville, MD, United States), following the manufacturer’s instructions. Degenerated primers (namely *qkia*F and *qkib*R) (**Table 1**) were designed based on highly conserved regions from the available sequence of zebrafish in GenBank. PCR amplification was made with 2 μL of RT reactions in a total volume of 50 μL and 2.5 U of Platinum Taq DNA Polymerase (Invitrogen). The PCR cycling conditions were set as follows: one cycle of 94°C for 4 min, 30 cycles of 94°C for 40 s, 52°C for 40 s, and 72°C for 60 s followed by one cycle of 72°C for 7 min. The PCR products were separated by agarose gel electrophoresis, and delivered to Shanghai Sangon Biotech Service Co., Ltd. (Shanghai, China) for sequencing. Sequencing was performed in both forward and reverse directions by an ABI PRISM^®^ 377 DNA automated sequencer (Applied Biosystems). The forward and reverse sequences were assembled using the SeqMan II software in DNASTAR package version 5.01, and the core fragment of *qkia* was obtained. Then, specific primers were designed for the 3′ and 5′ RACE according to this sequence.

**Table 1:** Primers used for the cDNA cloning of *qkia* and RT-PCR.

	Sequence (5′ → 3′)	Use
*qkia*-F	GGGGAGATGGAGGTGA	Used with *qkia*-R for RT-PCR of core fragment
*qkia*-R	CACCCGCTGGAGTTAC	Used with *qkia*-F
Oligo(dT)_16_AP	CTGATCTAGAGGTACCGGATCC(T)_16_	Synthesis of the first-strand cDNA for 3′ RACE
*qkia*,3-F1	CACCAACCCTCCATACC	Used with AP for first PCR of 3′ RACE
AP	CTGATCTAGAGGTACCGGATCC	Used with *qkia*, 3-F1 or *qkia*-R2
*qkia*,3-F2	CCAACCCTCCATACCAA	Used with RACE3-R for nested PCR of 3′ RACE
RACE3-R	AACAGCCACGCTCGCAGA	Used with *qkia*, 3-F2
*qkia*,5-R	AGAGCGTCACCACAGC	Synthesis of the first-strand cDNA for 5′ RACE
*qkia*,5-R1	AGAGCGTCACCACAGC	Used with Oligo(dT)16AP for first PCR of 5′ RACE
*qkia*,5-R2	AGGATTGTGAGCGTTTG’	Used with AP for nested PCR of 5′ RACE
q *qkia*-F	ATGATGGTCGGGGAGATG	Used with q *qkia*-R for RT-PCR of tissue distribution
q *qkia*-R	GAAGATGCCGCACAGGTT	Used with q *qkia*-F
EF1α-F	CTTCTCAGGCTGACTGTGC	Used with EF1α-R as internal standard
EF1α-R	CCGCTAGCATTACCCTCC	Used with EF1α-F

The 3′ end was amplified with a 3′-full RACE (rapid amplification of cDNA end) Core Set (Takara, Dalian, China) following the instructions. Firstly, 2 μg of total RNA was reverse-transcribed using Oligo(dT)_16_AP as the primer to obtain the first strand cDNA. Then, it was diluted to 1:10 with dH_2_O, and was used as a template for PCR. The cDNA was amplified by a specific forward primer *qkia*3-F1 and a reverse primer AP containing the anchor sequence. After this, a dilution (0.1%) of the original PCR was re-amplified using a reverse primer RACE3-R and a specific forward primer *qkia*3-F2. The nested PCR product was sequenced by the electrophoresis analysis aforementioned. The primers used were shown in **Table 1**.

The 5′ RACE was performed using the SMARTer^TM^ RACE cDNA Amplification Kit (Clontech). Reverse transcription of 2 μg total RNA was made with a specific reverse primer *qkia*-R to obtain the first strand cDNA. Then, the purified cDNA was used in the TdT-tailing reaction. Tailed cDNA was amplified by prime pairs *qkia*5-R1/Oligo (dT)_16_ AP. The first PCR product was diluted to 1:10 with dH_2_O, and was used as a template for the nested PCR, which was performed with AP and *qkia*5-R2. The nested PCR product was sequenced following the methods detailed above. The primers used were shown in **Table 1**.

### Sequence Alignment, Structure Prediction, and Phylogenetic Analysis of *qkia*

The full-length cDNA of *qkia* was assembled by the SeqMan II software in DNA Star Package (version 5.01). Then, the amino acids (AA) sequences were obtained by an online Open Reading Frame (ORF) Finder program^1^. The molecular weight (MW) and isoelectric point (PI) of the Qkia protein was both predicted using the Compute pI/Mw software at http://cn.expasy.org/tools/pi_tool.html. The NCBI Conserved Domain Search^2^ was used to predict the conserved functional sites. The secondary and three-dimensional (3D) structures of Qkia protein were predicted by the SABLE program^3^ and the SWISS-MODEL program^4^, respectively. Multiple alignments were generated by the Clustal program in DNA Star package (version 5.01). The phylogenetic analyses were carried out based on the AA sequences using the Neighbor-Joining algorithm method within the MEGA 4.0 program and the reliability of this estimated tree was evaluated by the bootstrap method with 1000 pseudo-replications.

### GTT and ITT

For GTT, four fish were randomly collected and slightly anesthetized in neutralized MS-222 (Sigma, St. Louis, MO, United States) at the concentration of 100 mg/L. Then they were sacrificed. Blood, brain, muscle and liver was quickly collected, which were used for time 0 h. The remaining 48 fish (the fish number was 56 in total) were equally divided into two groups, and individually weighed and intraperitoneally injected with either glucose [1.67 g glucose per kg body weight (BW)] or saline solution (16.7 ml/kg BW, the sham treatment) within 10 min (Li et al., 2016). A saline solution (0.9%) containing 100 mg glucose per ml was used for that purpose. After administration, fish were immediately transferred to 12 small tanks (100 L each) at a density of four fish per tank. The fish in each treatment were anesthetized and sampled at 1, 2, 4, 8, 12, and 24 h, respectively, after injection. One aquarium was used for each sampling time in order to minimize the stress due to sampling. Blood, brain, muscle, and liver was quickly collected. The blood was centrifuged at 2500 rpm at 4°C for 10 min. Then, both the supernatant and tissues were stored at -80°C for further analysis.

For ITT, the samples for time 0 h were obtained following the procedures detailed in GTT. Then, the remaining 84 fish were equally divided into three groups, and individually weighted and injected intraperitoneally with bovine insulin (0.052 mg/kg BW, Sigma, United States) (Jin et al., 2014), bovine glucagon (0.075 mg/kg BW, Shanghai Rongbai Biological Technology Co., Ltd., China) (Albalat et al., 2005) or saline solution (1.0 ml/kg BW, the sham treatment), respectively. A saline solution (0.9%) containing either 0.052 mg insulin per ml or 0.075 mg glucagon per ml was used for that purpose. Then, fish of each treatment were immediately transferred to seven small tanks at a density of four fish per tank with their blood, brain, muscle, and liver sampled at 1, 2, 4, 6, 8, 12, and 24 h, respectively, after injection following the procedures detailed in GTT. It should be mentioned here that glucagon was adopted in ITT as a supplement to investigate the transcriptional responses of *qkia* to different hormones in order to better understand the potential roles of *qkia* in glucose metabolism.

### Feeding Trial

After the GTT and ITT, another group of fish (average weight: 24.24 ± 0.05 g) were subjected to a feeding trial. Briefly, after acclimation, 160 fish were randomly and equally distributed into eight tanks (300 L each) at a stocking density of 20 juveniles per tank in a recirculating aquaculture system. Then, fish were “hand-fed to apparent” satiation three times daily (07:00, 12:00, and 17:00 h) for 12 weeks with one of two isonitrogenous (30% crude protein) diets (**Table 2**) containing either 31% (control diet) or 41% (high carbohydrate diet) nitrogen-free extract (Li et al., 2013, 2014). Each diet was tested in four tanks. During this period, a 12:12 h light: dark regime (07:00 to 19:00 h light period) was maintained by timed fluorescent lighting. Aeration was provided during the 24 h. Water temperature ranged from 26 to 28°C, pH from 7.1 to 7.4, and total ammonia-nitrogen was maintained below 0.2 mg/L. At the termination of the feeding trial, fish were starved for 24 h for gut clearance. Then, fish were slightly anesthetized. Four fish per tank were quickly collected for blood, brain, liver, and muscle, which were treated and stored following the procedures aforementioned.

**Table 2:** Formulation and proximate composition of the different experimental diets.

	Control	High carbohydrate (HC)
*Formulation* (*%*)		
Fish meal	8.00	8.00
Soybean meal	26.00	26.00
Rapeseed meal	17.00	17.00
Cottonseed meal	17.00	17.00
Fish oil	2.00	2.00
Soybean oil	2.00	2.00
Corn starch	12.00	25.00
Microcrystalline cellulose	13.00	0.00
Calcium biphosphate	1.80	1.80
Premix^1^	1.20	1.20
*Proximate composition (% air-dry basis*)		
Moisture	6.96	6.93
Crude lipid	5.93	5.94
Ash	8.46	8.78
Crude protein	29.82	30.23
Crude fiber	16.97	7.48
Nitrogen-free extract^2^	31.86	40.64
Energy (MJ/kg)	19.09	19.14

*Control, diet with 31% carbohydrate; HC, diet with 41% carbohydrate; ^1^Premix supplied the following minerals (g/kg of diet) and vitamins (IU or mg/kg of diet): CuSO_4_⋅5H_2_O, 2.0 g; FeSO_4_⋅7H_2_O, 25 g; ZnSO_4_⋅7H_2_O, 22 g; MnSO_4_⋅4H_2_O, 7 g; Na_2_SeO_3_, 0.04 g; KI, 0.026 g; CoCl_2_⋅6H_2_O, 0.1 g; Vitamin A, 900,000 IU; Vitamin D, 200,000 IU; Vitamin E, 4500 mg; Vitamin K_3_, 220 mg; Vitamin B_1_, 320 mg; Vitamin B_2_, 1090 mg; Vitamin B_5_, 2000 mg; Vitamin B_6_, 500 mg; Vitamin B_12_, 1.6 mg; Vitamin C, 5000 mg; Pantothenate, 1000 mg; Folic acid, 165 mg; Choline, 60,000 mg. ^2^Calculated by difference (100 – moisture – crude protein – crude lipid –ash – crude fiber).*

### Analysis of Proximate Composition and Plasma Glucose Levels

The proximate composition of diets and fish was ensured based on the standard AOAC method. Moisture was determined by oven drying at 105°C until constant weight. Crude protein content (nitrogen × 6.25) was measured by the micro-Kjeldahl method using an Auto Kjeldahl System (FOSS KT260, Switzerland). Crude lipid was determined by solvent extraction using the Soxtec System HT (Soxtec System HT6, Tecator, Sweden) and ash by combustion at 550°C for 4 h. Gross energy was determined using a Bomb Calorimeter (PARR 1281, Parr Instrument Company, Moline, IL, United States). Crude fiber was determined by the fritted glass crucible method using an automatic analyzer (ANKOM A2000i, Macedon, New York, NY, United States). Plasma glucose was measured using the glucose oxidase method (Asadi et al., 2009).

### Real-Time PCR

The mRNA expression of *qkia* was quantified by semi-quantitative RT-PCR. For tissue distribution, total RNA was extracted from 10 tissues and/or organs as aforementioned with the quantity, purity and integrity tested. Equal quantities of each total RNA (2 μg) (as template) and Oligo(dT)_18_ (as primer) were used to reverse-transcribe the respective RNA. The resulting first strand cDNA was diluted and used as template for PCRs. PCR reactions were conducted using the CFX96 Touch^TM^ Deep Well Real-Time PCR Detection System (Bio-Rad). Amplifications were performed in a reaction volume of 20 μL, containing 2 μL DNA sample, 10 μL 2 × SYBR green Real time PCR Master Mix (Takara, Dalian, China), 0.2 μL of each primer (*qkia*F2 and *qkia*R2 in **Table 1**) and 7.6 μL H_2_O. The PCR conditions were set as follows: 3 min at 95°C, followed by 45 cycles of 40 s at 95°C, and 40 s at 62°C. Each sample was run in triplicate. PCR reactions without the addition of the template were used as negative controls. At the end of the reaction, the fluorescent data were converted into *C*t values. Each transcript level was normalized to EF1α (Zhang et al., 2013) using the 2^-ΔΔC_T_^ method (Livak and Schmittgen, 2001). It should be mentioned that all the PCRs were highly specific and reproducible (0.998 > *R*^2^ > 0.983), and all primer pairs had equivalent PCR efficiencies (from 0.89 to 1.14).

Tissue *qkia* expressions of fish in the GTT, ITT and after the feeding trial were also measured following the procedures detailed above.

### Statistical Analysis

The data concerning the tissue distribution of *qkia* and its transcription as well as plasma glucose levels after high-carbohydrate feeding were analyzed by one-way ANOVA using the SPSS 16.0 for Windows software package, after testing the homogeneity of variances with the Levene test. Unlikely, data regarding plasma glucose levels and tissue *qkia* expressions after the intraperitoneal load of glucose, insulin, and glucagon were analyzed by two-way ANOVA for significant differences among treatment means based on sampling time, injection type and their interaction. If significant differences were observed (*P* < 0.05) in the interaction, each factor was further analyzed separately by one-way ANOVA. Specifically, data among different sampling time within each treatment was analyzed by one-way ANOVA. Data among the different treatments at each sampling time was also analyzed by one-way ANOVA. Significant differences among groups were determined by Tukey’s multiple range test. All data were presented as means ± SEM (standard error of the mean).

## Results

### Molecular Characterization of the *qkia* Gene

**FIGURE 1:**
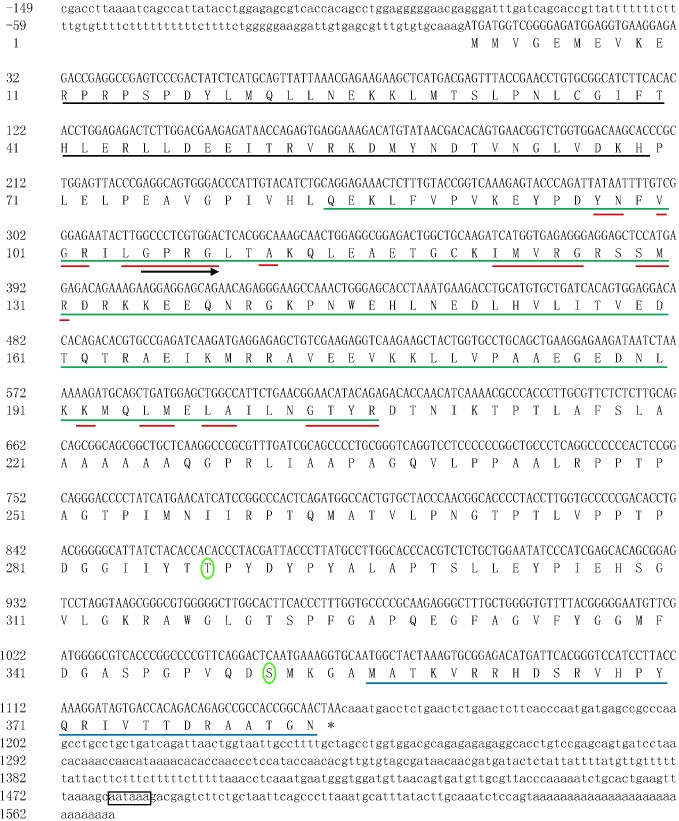
The nucleotide sequence of the *qkia* cDNA in blunt snout bream, and the deduced amino acid sequence. Uppercase letters indicate the translated region, whereas lowercase letters represent the untranslated region. Blank line indicates the STAR_dimer, green line reveals the SF1_like-KH, red line suggests the potential RNA-binding sites, and blue line represents the Quaking_NLS. Right arrow indicates the G-X-X-G motif. An asterisk suggests the termination codon. The phosphorylated sites are circled and the polyadenylation signal is boxed. The nucleotide sequence was submitted to NCBI GenBank, accession number: KY_444733.

In this study, the full-length cDNA encoding *qkia* (GenBank accession number: KY444733) was characterized from the liver of *M. amblycephala*. As was presented in **Figure 1**, the full-length cDNA covered 1,718 bp, with an ORF of 1,152 bp encoding 383 AA. The 5′-UTR covered 149 nt. An ATG initiation codon was observed 150 nt downstream of the 5′-start, and a TAA stop codon was present 418 nt upstream of the 3′-end. The 3′-UTR covered 417 nt including one AATAAA motifs, which represents putative polyadenylation signals (nt 1,479–1,484). The Qkia protein has a calculated MW of 42203.74 Da and PI of 8.34. This AA sequence contains several conserved functional sites, including one G-X-X-G motif (Gly105-Gly108), 10 RNA-binding sites (Tyr97-Asn98, Val100-Arg102, Leu104-Gly108, Ala111, Ile122-Gly126, Ser129-Arg131, Lys192, Leu195-Met196, Leu198-Ala199, and Gly203-Arg206), one STAR (signal transducer and activator of RNA)_dimer (Arg11-His69), one SF1_like-KH [Splicing factor 1 (SF1) K homology RNA-binding domain (KH)] (Gln84-Arg206) and one Quaking_NLS (Putative nuclear localization signal of quaking) (Met356-Asn383). Based on the results predicted by the online SABLE program, the secondary structure of this Qkia protein consists of 5 α-helices, 5 β-strands and 11 C-coils (Supplementary Figure S1). The 3D model constructed was shown in Supplementary Figure S2.

**FIGURE 2:**
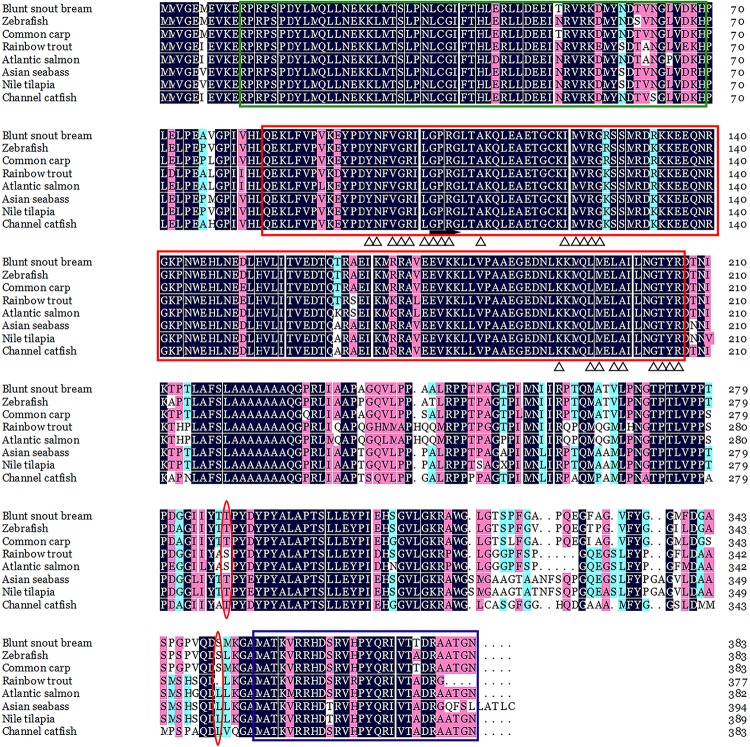
ClustalW alignment of the deduced AA sequence of the *qkia* protein from *Megalobrama amblycephala* and other organisms. Identical amino acids are marked in black and homologous amino acids in pink. The green, red and purple box indicate the STAR_dimer, SF1_like-KH and Quaking_NLS, respectively. Arrow and triangle indicates the G-X-X-G motif and potential RNA-binding sites, respectively. The phosphorylation sites are marked in red circle. The accession numbers for the sequences are as follows: blunt snout bream *M. amblycephala* (KY_444733), zebrafish (*Danio rerio*) (NP_571299.1), common carp (*Cyprinus carpio*) (XP_018921881.1), rainbow trout (*Oncorhynchus mykiss*) (CDQ_57984.1), Atlantic salmon (*Salmo salar*) (XP_013981550.1), Asian seabass *Lates calcarifer* (XP_018518983.1), Nile tilapia (*Oreochromis niloticus*) (XP_005464024.3), channel catfish (*Ictalurus punctatus*) (X _017331508.1).

In addition, the Qkia protein of blunt snout bream has a high similarity, and shows similar structural features to that of the other piscine species (**Figure 2**). Complete AA sequence alignment showed the best identity with common carp (the homology is 97%), and less identity with rainbow trout (*Oncorhynchus mykiss*) (the homology is 84%).

### Phylogenetic Analysis of the Qkia Protein

**FIGURE 3:**
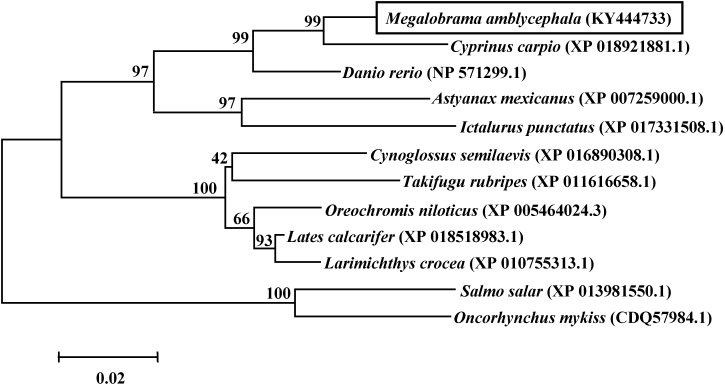
Phylogenetic tree based on the AA sequences of *qkia* made with the MEGA 3.1 software using the neighbor-joining method. The distance matrix was calculated using Kimara’s two-parameter model. The numbers represent bootstrap percentages. The topology was tested using bootstrap analyses (1,000 replicates). GenBank Accession numbers of the sequences are indicated on figure.

The phylogenetic tree among twelve piscine species based on the AA sequences of the Qkia protein was presented in **Figure 3**. Qkia of blunt snout bream (*M. amblycephala*) showed a close phylogenetic relationship with that of several Cyprinomorpha species, like common carp (*Cyprinus carpio*), zebrafish (*Danio rerio*) and the Mexican blind cavefish (*Astyanax mexicanus*) sharing high bootstrap values (97–99%). Conservation of Qkia was also evident from the high bootstrap values observed between Qkia of blunt snout bream and that of other fish, like the Siluriformes species: channel catfish (*Ictalurus punctatus*), the Percomorpha fish: Nile tilapia (*Oreochromis niloticus*), and the Salmoniformes species: Atlantic salmon (*Salmo salar*) and rainbow trout (*Oncorhynchus mykiss*). The phylogenetic relationship based on the AA sequences of Qkia agreed with the traditional classification.

### Tissue Distribution of *qkia* mRNA

**FIGURE 4:**
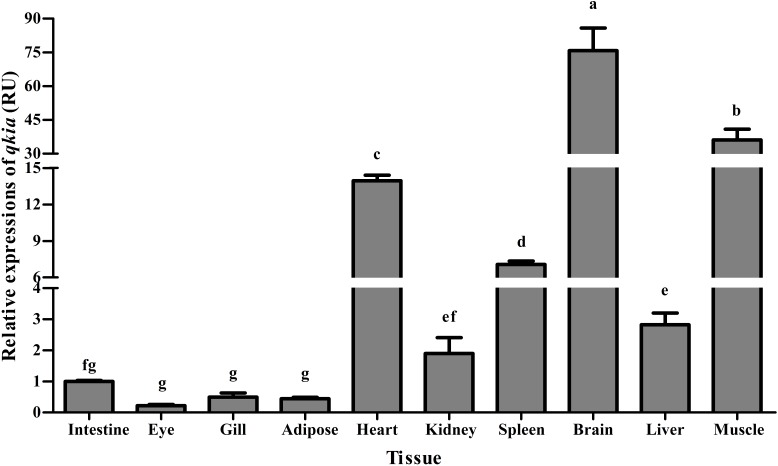
Relative expressions of *qkia* in various tissues of *M. amblycephala*. For tissue expression, data are referred to the values (Relative units, RU) found in the intestine. Each data represents the mean of eight replicates. Bars assigned with different superscripts are significantly different (*P* < 0.05).

As was shown in **Figure 4**, the *qkia* mRNA was detected in all examined tissues. The highest transcription was found in brain followed by muscle and heart. The transcriptional levels in liver, spleen, and kidney were all moderate, whereas relatively low values were detected in the mesenteric adipose tissue, gill and eye.

### Plasma Glucose Levels and Tissue *qkia* Expressions in the GTT

**FIGURE 5:**
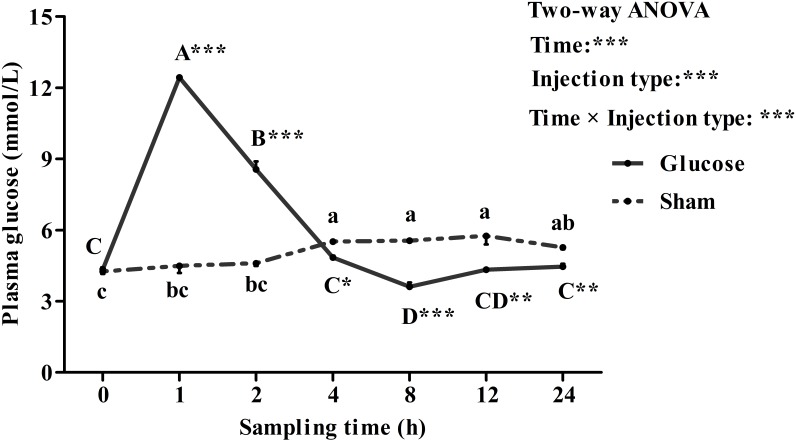
Plasma glucose levels of blunt snout bream subjected to a glucose load. Each data represents the mean of four replicates. Significant differences (*P* < 0.05) among sampling times within each treatment are indicated by different letters (lower case for sham, upper case for glucose). ^∗^Indicates a significant difference (*P* < 0.05) between the two treatments at each sampling time. ^∗^*P* < 0.05, ^∗∗^*P* < 0.01, ^∗∗∗^*P* < 0.001; ns, not significant.

Plasma glucose levels in blunt snout bream after a glucose load were shown in **Figure 5**. Plasma glucose concentration were significantly (*P* < 0.001) affected by sampling time, injection type and their interaction. The glucose loading resulted in a significantly (*P* < 0.05) increased plasma glucose level with the highest value being recorded at 1 h after injection. Thereafter, it decreased significantly (*P* < 0.05) to the basal value at 12 h and then remained constant until the end of the trial. Plasma glucose levels of the sham treatment showed no statistical difference (*P* > 0.05) during the first 2 h. Then it increased significantly (*P* < 0.05) from 2 to 4 h, and remained constant thereafter. In terms of injection type, plasma glucose levels of fish receiving glucose were significantly (*P* < 0.001) higher than that of fish offered saline solution. In addition, plasma glucose levels were significantly (*P* < 0.001) affected by the interaction between sampling time and injection type with significant (*P* < 0.05) differences observed at times 1, 2, 8, 12, and 24 h between the two groups.

**FIGURE 6:**
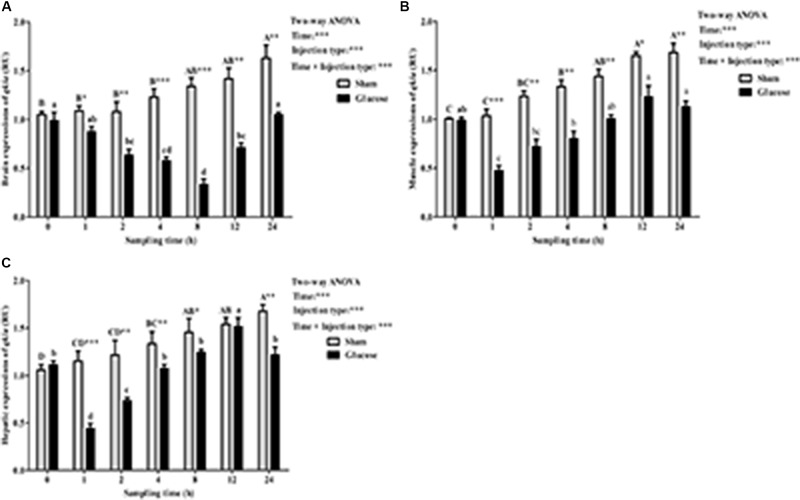
Relative expressions of *qkia* in the brain **(A)**, muscle **(B)**, and liver **(C)** of *M. amblycephala* after the glucose loading. Data are referred to the value (Relative units, RU) obtained in the corresponding tissue of the sham treatment solution at 0 h. Each data represents the mean of four replicates. Significant differences (*P* < 0.05) among sampling times within each treatment are indicated by different letters (lower case for glucose, upper case for sham). ^∗^Indicates a significant difference (*P* < 0.05) between the two treatments at each sampling time. ^∗^*P* < 0.05, ^∗∗^*P* < 0.01, ^∗∗∗^*P* < 0.001; ns, not significant.

As was shown in **Figure 6**, after the glucose load, the *qkia* transcriptions in brain (**Figure 6A**), muscle (**Figure 6B**), and liver (**Figure 6C**) were all significantly (*P* < 0.001) affected by sampling time, injection type and their interaction. The glucose administration resulted in a significant (*P* < 0.05) decrease of *qkia* expression in both liver and muscle with the lowest value being recorded at 1 h after injection. Then, they both increased significantly (*P* < 0.05) to the highest value at 12 h. However, brain *qkia* expressions decreased significantly (*P* < 0.05) with the lowest value being recorded at 8 h after the glucose administration. Then it gradually increased to the basal value at 24 h. As for the sham treatment, *qkia* expressions gradually increased in these tissues during the whole sampling period. In terms of injection type, *qkia* expression of fish injected glucose was significantly (*P* < 0.001) lower than that of the sham treatment. In addition, tissue *qkia* expressions were significantly (*P* < 0.001) affected by the interaction between sampling time and injection type with significant (*P* < 0.05) differences observed at times 1–24 h in these tissues (except for the time point of 12 h in liver) between the two groups.

### Plasma Glucose Levels and Tissue *qkia* Expressions in the ITT

**FIGURE 7:**
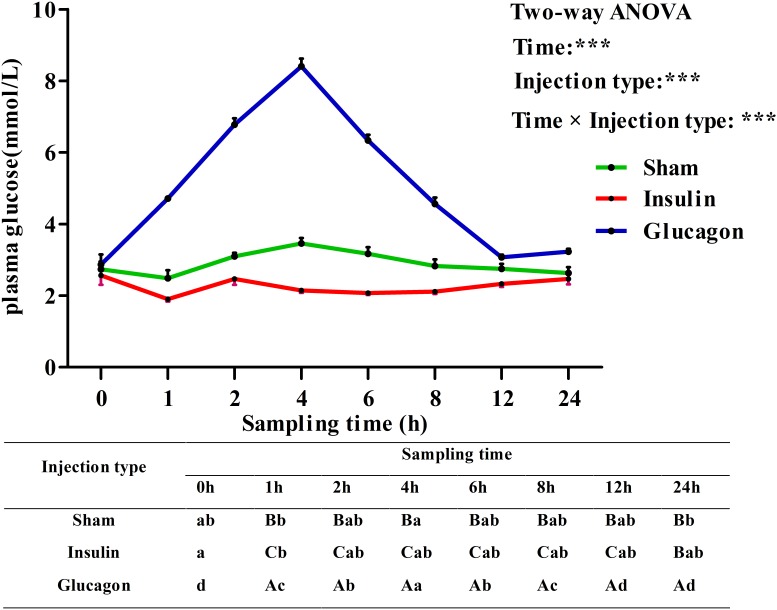
Plasma glucose levels of blunt snout bream subjected to the insulin and glucagon load. Each data represents the mean of four replicates. Different lower-case letters indicate significant differences (*P* < 0.05) at different time points within each treatment, whereas different capital letters indicate significant differences (*P* < 0.05) at each sampling point among different treatments. ^∗^*P* < 0.05, ^∗∗^*P* < 0.01, ^∗∗∗^*P* < 0.001; ns, not significant.

Plasma glucose levels in blunt snout bream after ITT were shown in **Figure 7**. Plasma glucose levels were significantly (*P* < 0.001) affected by sampling time, injection type and their interaction. The insulin load resulted in a significant (*P* < 0.05) decrease of plasma glucose levels with the lowest value being recorded at 1 h, then it showed no statistical difference (*P* > 0.05) thereafter. The glucagon load resulted in a sharp increase of glycemic levels with the highest value being recorded at 4 h, then it decreased significantly (*P* < 0.05) to the basal level at 12 h. Saline injection led to a slight (*P* > 0.05) decrease of plasma glucose levels during the first 1 h. Then it increased significantly (*P* < 0.05) from 1 to 4 h with the maximum value being attained at 4 h. Thereafter, it gradually returned to the basal value with further increasing time. In terms of injection type, the glycemic values of fish receiving insulin were significantly lower (*P* < 0.001) than those of fish injected glucagon and saline solution during the whole sampling period. Furthermore, plasma glucose levels were significantly (*P* < 0.001) affected by the interaction between sampling time and injection type with significant (*P* < 0.05) differences observed at times 1–24 h among the three groups.

**FIGURE 8:**
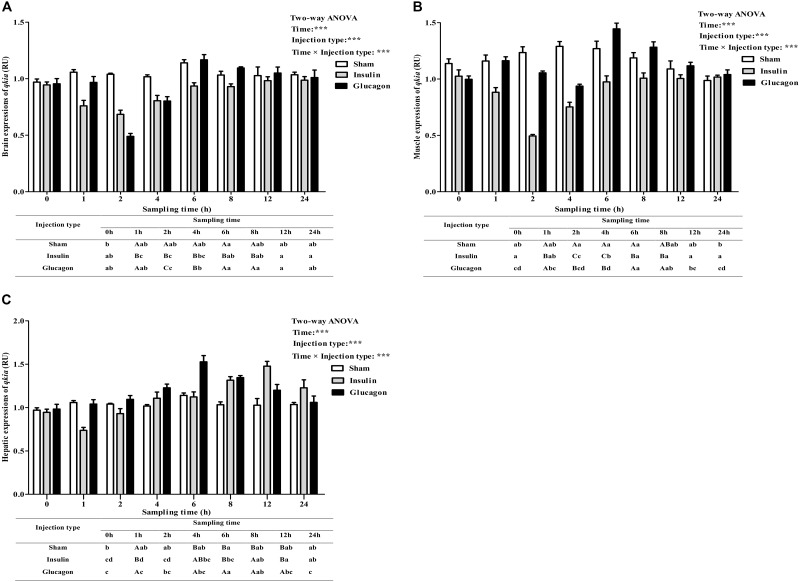
Relative expressions of *qkia* in the brain **(A)**, muscle **(B)**, and liver **(C)** of *M. amblycephala* after the insulin and glucagon loading. Data are referred to the value (Relative units, RU) obtained in the corresponding tissue of the sham treatment at 0 h. Each data represents the mean of four replicates. Different lower-case letters indicate significant differences (*P* < 0.05) at different time points within each treatment, whereas different capital letters indicate significant differences (*P* < 0.05) at each sampling point among different treatments. ^∗^*P* < 0.05, ^∗∗^*P* < 0.01, ^∗∗∗^*P* < 0.001; ns, not significant.

As was shown in **Figure 8**, after the insulin and glucagon administration, the *qkia* expressions in brain (**Figure 8A**), muscle (**Figure 8B**), and liver (**Figure 8C**) were all significantly (*P* < 0.001) affected by sampling time, injection type and their interaction. The insulin administration resulted in a significant (*P* < 0.05) decrease of *qkia* expression in brain, muscle and liver with the lowest value being recorded at 2 h in brain and muscle and 1 h in liver, respectively. Subsequently, the expressions in these tissues all increased significantly (*P* < 0.05) to the basal value. The glucagon load induced a significant (*P* < 0.05) increase of *qkia* expression in liver during the first 6 h. Then it gradually decreased to the basal value. However, brain and muscle *qkia* expressions decreased significantly (*P* < 0.05) at 2–4 h after glucagon administration. Then they both increased significantly (*P* < 0.05) with the highest value being recorded at 6 h, and gradually reduced to the basal value thereafter. As for the sham treatment, the *qkia* expressions increased significantly (*P* < 0.05) in both brain and liver during the first 6 h, thereafter it showed no statistical difference (*P* > 0.05). Muscle *qkia* expression also showed a similar trend from 0 to 6 h, whereas no statistical difference (*P* > 0.05) thereafter. In terms of injection type, *qkia* expression of fish receiving insulin were significantly lower (*P* < 0.001) than those of fish injected glucagon and saline solution (except for the liver). In addition, tissue *qkia* expressions were significantly (*P* < 0.001) affected by the interaction between sampling time and injection type with significant (*P* < 0.05) differences observed at times 1–12 h (except for the time point of 12 h in brain and muscle) among the three groups.

### Plasma Glucose Levels and Tissue *qkia* Expressions After the Feeding Trial

**FIGURE 9:**
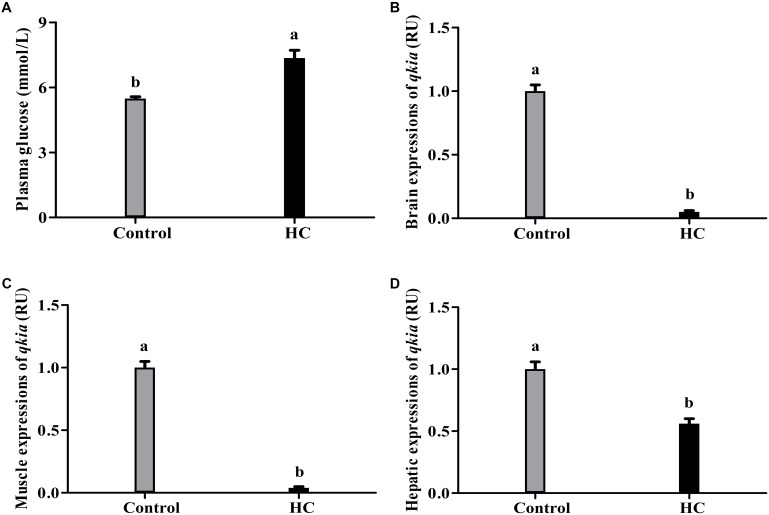
Plasma glucose levels **(A)** and relative expressions of *qkia* in the brain **(B)**, muscle **(C)**, and liver **(D)** of *M. amblycephala* subjected to different dietary carbohydrate levels. For mRNA expressions, data are referred to the value (Relative units, RU) obtained in the corresponding tissue of fish fed the control diet. Each data represents the mean of four replicates. Values with the different lower-case letters are significantly different among groups (*P* < 0.05).

As can been from **Figure 9**, high-carbohydrate feeding induced a significant (*P* < 0.05) increase of plasma glucose levels (**Figure 9A**). However, the transcriptional levels of *qkia* significantly (*P* < 0.05) decreased in the brain (**Figure 9B**), muscle (**Figure 9C**), and liver (**Figure 9D**) after high-carbohydrate intake.

## Discussion

In this study, a full-length cDNA coding *qkia* was cloned from the liver of a herbivorous freshwater carp blunt snout bream. Based on the results of sequence alignments, the functional sites are all highly conserved in the species detected except for some minor AA changes. This high similarity strongly suggested that this sequence corresponded to the functional *qkia*. As for the STAR_dimer, the Thr51 and 61 (blunt snout bream numbering) is replaced by Asn51 and Ser61, respectively, in zebrafish. This difference might partly lead to the variations of the functions of *qkia* among different species, since the STAR_dimer participates in the binding of STAR proteins to bipartite RNA (Beuck et al., 2012), thus maintaining the normal function of *qkia* (Chen and Richard, 1998). For phosphorylation sites, the Ser 351 and Thr288 (blunt snout bream numbering) are replaced by Leu357 and Ser 289 (tilapia numbering) respectively, in other piscine species. These replacements might result in the large changes of the transcriptional activity of QKI among different species, since phosphorylation is an important post-translational modification of proteins, which can greatly affect their functions (Besant and Attwood, 2009). In addition, some minor AA differences were also observed in the SF1_like-KH between mammals and fish, as might also lead to the variations of the functions of *qkia* among different species. This was supported by the fact that, as a prevalent RNA binding domain, the SF1_like-KH could specifically recognize the intron branch point sequence in the pre-mRNA transcripts (Liu et al., 2001), as is closely involved in the regulation of the activity of RBPs (Zorn et al., 1997). Furthermore, a large difference was observed in the AA sequence of the Quaking_NLS. A total of 28 AA was characterized in the Quaking_NLS of fish (blunt snout bream for example), whereas only 7 were found in mouse and *Drosophila* (Wu et al., 1999). This might also result in the large variations of the transcriptional activity of *qkia* among different species, since the Quaking_NLS is the very C-terminal region of quaking proteins that is purported to be the nuclear localization signal (Lobbardi et al., 2011).

In *M. amblycephala*, the *qkia* mRNA was detected in all the organs/tissues examined. According to previous studies, QKI could participate in the post-transcriptional regulation of forkhead transcription factor 1 (Foxo1) (transcription factor of energy metabolism), thus playing an important role in the energy metabolism in various tissues and/or organs in mammals (Guo et al., 2014; Lu, 2014; Yu et al., 2014). In addition, a tissue-specific expression pattern of *qkia* was also detected. The predominant expression was found in brain followed by muscle and heart, as was similar to the results found in mammals and other piscine species (Ebersole et al., 1996; Tanaka et al., 1997; Kondo et al., 1999). This result was not surprising due to the fact that fish brain has high glucose utilization rates per unit mass (Washburn et al., 1992). In addition, previous studies showed that muscle is the main site of glucose uptake by glucose transporters (GLUTs), and heart also plays an important role in glucose uptake (Blasco et al., 1996; Deck et al., 2017). Hence, these findings probably suggested that *qkia* may also play an important role in the energy metabolism of fish. The remarkable expressions found in liver, spleen, and kidney were also justifiable, since (1) a previous study has showed that *qki* is closely involved in the regulation of hepatic energy metabolism in mice (Lu, 2014); (2) both spleen and kidney play an important role in the glucose metabolism of fish (Hall et al., 2009). In addition, the transcription of *qkia* was also detected in the eye, intestine and the mesenteric adipose tissue of *M. amblycephala*. This was quite different from that of mammals, who exhibited a relatively low mRNA level of *qki* in these tissues (Kondo et al., 1999). This difference might partly suggest the distinct mechanisms by which different animal species use glucose for energy purposes. Furthermore, the presence of *qkia* in gill was also reasonable, since gill plays a key role in maintaining the oxygen demand, which can regulate adenosine triphosphate (ATP) turnover, thus enhancing the oxygen-carrying capacity of blood (Wells et al., 1989). Together, these findings probably suggested that *qkia* may play a crucial role in the energy metabolism (especially the glucose metabolism) of fish. However, further studies are warranted to elucidate this.

In this study, the glucose load resulted in a drastically increased plasma glucose level with the highest value being gained at 1 h. Time to reach the highest glucose value in blunt snout bream was similar to that of several herbivorous and omnivorous species, such as tilapia *Oreochromis niloticus* × *O. aureus* (Lin et al., 1995) and common carp (Furuichi and Yone, 1981) receiving the same glucose dose, but was faster than that of most carnivorous fish like red sea bream *Chrysophrys* major, yellowtail *Seriola quinqueradiata* (Furuichi and Yone, 1981), European sea bass *Dicentrarchus labrax* (Enes et al., 2011), turbot *Scophthalmus maximus*, and snapper *Pagrus auratus* (Garcia-Riera and Hemre, 1996; Booth et al., 2006), which needed 2–3 h to reach the glucose peak. According to previous study, differences in timing required to obtain glucose peak was mainly ascribed to species differences although that of fish size and experimental conditions cannot be discarded (Legate et al., 2001; Moon, 2001; Polakof et al., 2012). In addition, time to clear the glucose load in blunt snout bream (4–6 h) was similar to that of omnivorous fish like tilapia (6 h) and common carp (5 h) (Furuichi and Yone, 1981; Chen et al., 2017), but was much faster than that of the carnivorous species (12–24 h) (Hemre et al., 1995; Booth et al., 2006; Enes et al., 2011). The results indicated that, being a herbivorous freshwater fish, blunt snout bream has a higher capacity to clear blood glucose compared with most carnivorous species. This may be ascribed to the fact that herbivorous fish have a higher glucose phosphorylation capacity in peripheral tissues (muscle and fat tissues) and a stronger suppression of hepatic gluconeogenesis (Kirchner et al., 2005; Polakof et al., 2012). Furthermore, plasma glucose levels of fish receiving saline solution slightly increased, as might be attributed to the handling stress caused by injection or the sampling procedures (Enes et al., 2012; Chen et al., 2017). It is also worth noting that significant difference was observed in plasma glucose levels at 4–24 h between the saline treatment and fish injected glucose. This might be ascribed to an enhancement of gluconeogenesis in fish in an attempt to maintain glucose homeostasis. Similar result was also found in white sea bream *Diplodus sargus* after a glucose load (Enes et al., 2012).

In the present study, the glucose load resulted in a remarkable down-regulation of *qkia* in the brain, liver, and muscle of fish. This probably suggested that *qkia* might play an important role in the glucose metabolism (especially the gluconeogenesis) of fish. This was supported by the fact that glucose administration generally induces an increase of plasma insulin levels, which significantly stimulates GLUTs expressions, and also increases the insulin-like growth factor-I (IGF-I) levels (Planas et al., 2000; Diaz et al., 2007, 2009; Enes et al., 2011). This consequently promotes glucose uptake, glycogen synthesis and lipogenesis, while depressing gluconeogenesis (Bouraoui et al., 2010; Enes et al., 2012). In addition, in this study, the glucose injection resulted in a prompt decrease of *qkia* expression in the liver and muscle during the first 1 h, which was much shorter than that (8 h) in the brain. This may be due to the fact that glucose injection usually leads to a prompt increase of liver glycogen by stimulating glycogen synthetase (GS) expressions (Hemre et al., 1991; Peres et al., 1999; Kamalam et al., 2017). Furthermore, the glucose administration also promptly enhanced the glucose phosphorylation capacity in muscle by increasing hexokinase (HK) activities (Kirchner et al., 2005). Unlikely, the brain of fish might perceive the glucose deriving from intraperitoneal injection slowly compared with the intraperitoneal tissues, leading to a delayed glucose-stimulated response. In addition, the brain of fish could meet its glucose demand through the breakdown of local glycogen independently of changes in circulating glucose levels, as might also lead to a delayed glucose response (Polakof et al., 2012). Furthermore, in this study, *qkia* expressions increased with the increasing sampling time after the initial decrease, as was in line with the results of plasma glucose levels observed in the GTT. This might be a metabolic adjustment of fish to keep normoglycemia through the enhancement of gluconeogenesis (Polakof et al., 2012). This was supported by the fact that *qkia* plays an important role in gluconeogenesis through the mediation of Foxo1 via post-transcriptional regulation (Yu et al., 2014). However, this was obtained in mammals. Whether this also holds true in aquatic species is still unknown, as warrants further studies. Furthermore, in terms of injection type, the *qkia* expression of fish receiving glucose was remarkably lower than that of the sham treatment. Generally, high exogenous glucose could enhance the glycolysis of fish, but inhibits the gluconeogenesis (Caseras et al., 2002; Kamalam et al., 2012). This again suggested that *qkia* might participate in the gluconeogenesis of fish.

In the present study, plasma glucose levels drastically decreased at 1 h after insulin administration. This may be due to the fact that insulin could promote glucose uptake by stimulating GLUT in peripheral tissues, and activates glycogen synthesis, but inhibits gluconeogenesis in fish (Sundby et al., 1991; Caruso and Sheridan, 2011). This inevitably led to the decrease of plasma glucose. In addition, time to reach the glucose minimum in blunt snout bream was faster than that of most carnivorous species (Jin et al., 2014, 2017; Deck et al., 2017), which required 3–9 h to attain the minimum value. This result might be ascribed to the facts that herbivorous fish has: (1) a higher capability of glycogen synthesis in muscle (Polakof et al., 2010); and (2) a stronger inhibition of hepatic gluconeogenesis than most carnivorous species (Navarro et al., 2006). In addition, plasma glucose returned to the baseline at 12 h after insulin administration. This was similar to the results obtained in gibel carp *Carassis auratus gibelio* (within 12 h) injected the same insulin dose (Jin et al., 2017), but was different with that of some carnivorous species like rainbow trout (Capilla et al., 2002), which required more than 24 h. After glucagon administration, the peak value of plasma glucose was obtained at 4 h. This was similar to that of common carp (Sugita et al., 2001), rainbow trout (Magnoni et al., 2001) and Japanese eel *Anguilla japonica* (Inui and Yokote, 1977), which required 2–3 h to reach the glucose peak after glucagon injection. This result may be explained by the fact that glucagon could stimulate glucose production of liver by binding glucagon receptor and activating its intracellular signal pathways (Chow et al., 2004; Yan et al., 2016), thus inevitably resulted in an increase of plasma glucose. Then, the glucose levels gradually returned to the normal value at 12 h, as was similar to the results obtained in Japanese eel (Inui and Yokote, 1977). In addition, in terms of injection type, plasma glucose level of fish injected with glucagon was significantly higher than that of fish received insulin. This might be due to the fact that glucagon is quite effective in elevating the concentration of glucose in the blood by promoting gluconeogenesis and glycogenolysis (Magnoni et al., 2001). Unlikely, insulin exerts opposite effects on circulating glucose levels through the stimulation of glycogenolysis as well as lipogenesis and concomitantly an inhibition of gluconeogenesis (Cheng et al., 2010).

In this study, both insulin and glucagon administration resulted in a prompt change of *qkia* at the transcriptional level in brain, muscle, and liver, indicating that *qkia* is quite sensitive to exogenous insulin and glucagon. The insulin injection resulted in a prompt decrease of *qkia* expression in the brain, muscle and liver, whereas the opposite was true after glucagon load. This suggested that *qkia* might participate in the gluconeogenesis and/or lipogenesis of fish, since insulin could depress the gluconeogenesis, glycogenolysis and lipolysis of fish, while glucagon is the major counterpart to insulin (Plisetskaya and Mommsen, 1996). In addition, insulin injection led to an immediate decrease of *qkia* expression in liver during the first 1 h, which was much shorter than that in the brain and muscle. This may be due to the fact that the muscle and brain of fish might be is less sensitive to exogenous insulin than liver, thus leading to a delayed insulin-stimulated response in both tissues (Caruso and Sheridan, 2011). After the initial decrease, *qkia* expressions gradually increased with increasing sampling time after insulin load. However, the opposite was true after glucagon administration. This again suggested that *qkia* might be closely involved in the gluconeogenesis of fish. Furthermore, in terms of injection type, the *qkia* expression of fish receiving insulin was lower than that of fish subjected to the glucagon load. This again reinforced the assumption that *qkia* might participate in the gluconeogenesis and/or lipogenesis of fish. This was supported by the following facts that (1) insulin could trigger a rapid and large glucose uptake by peripheral tissues, activating glycogen synthesis and lipogenesis, while suppressing gluconeogenesis (Polakof et al., 2010); and (2) glucagon could stimulate glycogenolysis, gluconeogenesis, and lipolysis (Magnoni et al., 2001).

In the present study, fish fed high-carbohydrate diets obtained relatively high plasma glucose levels. This indicated that high-carbohydrate intake induced a hyperglycemia state of blunt snout bream. In addition, the *qkia* expressions in brain, muscle, and liver were all significantly down-regulated in fed high carbohydrate diets. This was in line with the fact that high-carbohydrate feeding usually depresses the gluconeogenic capability of fish, as again suggested that *qki*a might play an important role in the glucose metabolism (especially the gluconeogenesis) of fish. According previous studies, the hyperglycemia state in fish induced by high-carbohydrate intake could stimulate insulin synthesis and release (Kamalam et al., 2017; Xu et al., 2018). This might inevitably result in the increased glucose disposal in peripheral tissue and the enhanced glycolysis and glycogenesis coupled with the suppression of gluconeogenesis (Polakof et al., 2012). In addition, excessive carbohydrate may be converted into lipids by enhancing the lipogenesis of blunt snout bream but depress the gluconeogenesis and glycogenolysis (Prisingkorn et al., 2017; Xu et al., 2018). However, further studies concerning the potential mechanisms by which *qkia* regulate the glucose metabolism of fish are needed to elucidate this.

## Conclusion

The present data suggested that the *qkia* gene of blunt snout bream shared a high similarity with that of the other vertebrates with the functions sites highly conserved. A graded tissue-specific expression pattern of *qkia* was also observed with high abundance observed in brain, muscle, and heart. Glucose and insulin administration, as well as high-carbohydrate feeding, remarkably down-regulated the transcriptions of *qkia* in brain, muscle, and liver, whereas the opposite was true after the glucagon load. These findings suggested that *qkia* might play an important role in the glucose metabolism of fish. However, further studies exploring the potential mechanisms are warranted to elucidate this.

## Author Contributions

X-FL and W-BL conceived and designed the experiment. CX and D-DZ analyzed the data. X-FL, H-JS, CX, B-KW, and LZ performed the experiments and contributed reagents/materials/analysis tools. X-FL and H-JS wrote the paper. All authors read and approved the final version of the manuscript.

## Conflict of Interest Statement

The authors declare that the research was conducted in the absence of any commercial or financial relationships that could be construed as a potential conflict of interest.

## Footnotes

1 http://www.ncbi.nlm.nih.gov/gorf/gorf.html

2 https://www.ncbi.nlm.nih.gov/Structure/cdd/wrpsb.cgi

3 http://sable.cchmc.org/

4 http://swissmodel.expasy.org/
