# Supplementary material for: Molecular Characterization of the RNA-Binding Protein Quaking-a in Megalobrama amblycephala: Response to High-Carbohydrate Feeding and Glucose/Insulin/Glucagon Treatment
Source: Front Physiol. 2018 Apr 24;9:434. doi: 10.3389/fphys.2018.00434 (PMC5928497; doi:10.3389/fphys.2018.00434)
Supplement: Supplementary file 1 [file Presentation_1.PDF]

Supplementary Material

Molecular Characterization of the RNA-binding Protein Quaking-a in *Megalobrama amblycephala*: Response to High-carbohydrate Feeding and Glucose/Insulin/Glucagon Treatment

Hua-Juan Shi, Wen-Bin Liu, Chao Xu, Ding-Dong Zhang, Bing-Ke Wang, Li Zhang and Xiang-Fei Li*

Key Laboratory of Aquaculture Nutrition and Feed Science of Jiangsu Province, College of Animal Science and Technology, Nanjing Agricultural University, No.1 Weigang Road, Nanjing 210095, People's Republic of China.

*Corresponding Author: Professor Xiang-Fei Li, E-mail:xfli@njau.edu.cn.

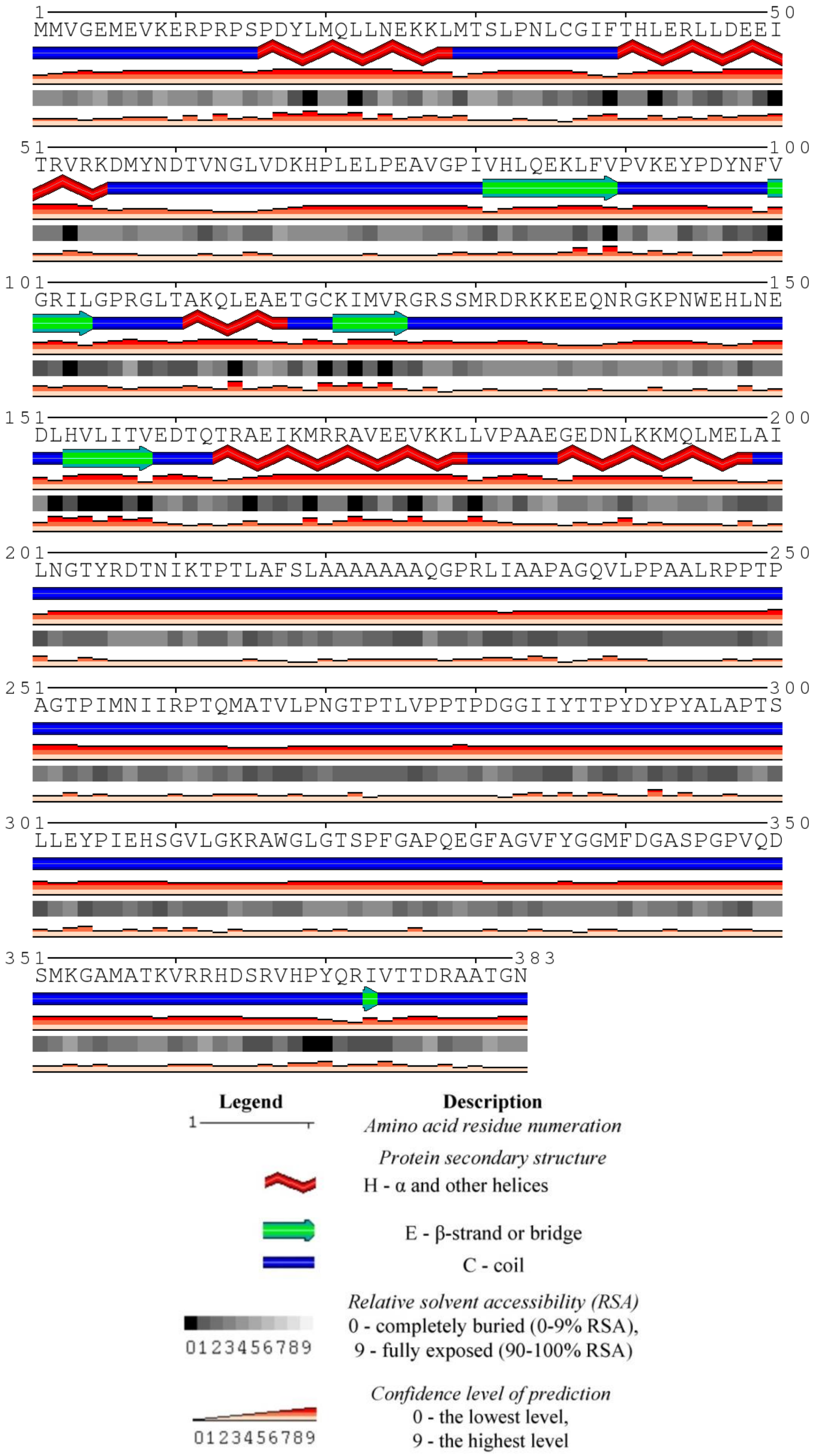

Figure 1. The secondary structure of the *qkia* protein in *Megalobrama amblycephala* constructed using the SABLE program.

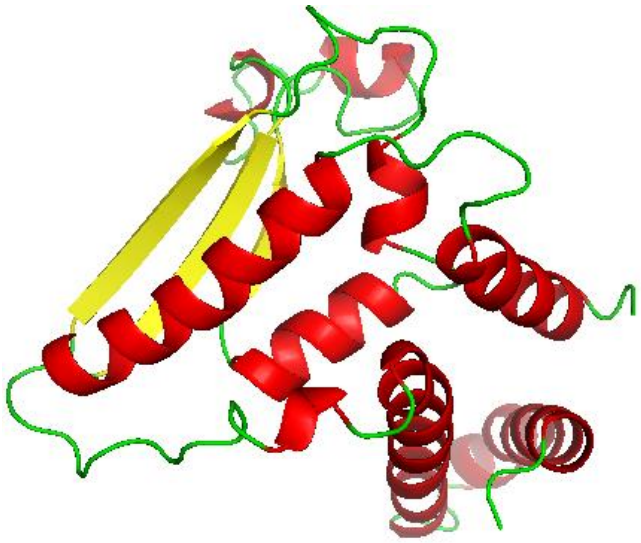

Figure 2. The 3D structure of the *qkia* protein in *Megalobrama amblycephala* constructed by the SWISS-MODEL.
